# Data on likelihood ratios of two-person DNA mixtures interpreted using semi- and fully continuous systems

**DOI:** 10.1016/j.dib.2019.104455

**Published:** 2019-09-04

**Authors:** Jae Joseph Russell B. Rodriguez, Jo-Anne Bright, Jazelyn M. Salvador, Rita P. Laude, Maria Corazon A. De Ungria

**Affiliations:** a DNA Analysis Laboratory, Natural Sciences Research Institute, College of Science, University of the Philippines Diliman, Quezon City, 1101, Philippines; b Genetics and Molecular Biology Division, Institute of Biological Sciences, College of Arts and Sciences, University of the Philippines Los Baños, Laguna, 4031, Philippines; c Institute of Environmental Science and Research Ltd., Mt. Albert Science Centre, Auckland, New Zealand

**Keywords:** DNA mixtures, Likelihood ratios, Post-coital samples, Continuous systems, Probabilistic systems

## Abstract

In the paper, “Probabilistic approaches to interpreting two-person DNA mixtures from post-coital specimens” [1], we analysed 102 two-person DNA samples from simulated mixtures and male-female and male-male post-coital specimens. We report here data on profile characteristics of these samples and likelihood ratios (LRs) generated using semi- and fully continuous systems. Both log_10_ LRs from true and non-contributor tests are presented. These data may supplement studies comparing performance of different probabilistic systems for DNA evidence interpretation.

**Table:** Specifications Table

Subject area	*Genetics*
More specific subject area	*Forensic Genetics*
Type of data	*Tables*
How data was acquired	*GeneMapper® ID-X v.1.2, LRmix Studio v.2.1.3, STRmix^TM^ v.2.5.11*
Data format	*Raw and Analysed*
Experimental factors	*Simulated two-person mixtures at different contributor ratios, various post-coital samples extracted using three DNA extraction procedures*
Experimental features	*Likelihood ratios for true and non-contributors to the two-person mixtures were generated using semi- and fully continuous systems for DNA interpretation.*
Data source location	*Quezon City, Philippines*
Data accessibility	*Raw and analysed data are presented in this article and attached as*Supplementary Material.
Related research article	J.J.R.B. Rodriguez, J.A. Bright, J.M. Salvador, R.P. Laude, M.C.A. De Ungria, Probabilistic approaches to interpreting two-person DNA mixtures from post-coital specimens, Forensic Sci. Int. 300 (2019) 157–163. https://doi.org/10.1016/j.forsciint.2019.04.037 0379-0738[1]

**Table:** 

**Value of the data** • The mixture profile characteristics of samples vis-à-vis corresponding likelihood ratios (LRs) can be used to investigate how the LR is affected by factors such as the number of drop-outs, average peak height, and mixture proportion of the person-of-interest (POI). • The LRs presented can add to data comparing performance of different software for mixture interpretation. • The dataset can be used in inter-laboratory comparisons employing different mixture interpretation strategies.

## 1 Data

Data on the quality of two-person DNA samples from simulated mixtures and post-coital samples are presented in Table 1. These mixtures were analysed using the LRmix Studio [2] and STRmix™ [3]. We present LRs from true contributor tests conditioned on the presence of a known contributor (*H1* true LRs) (Table 2). We further report a summary of non-contributor tests (Table 3) and attach a list of all non-zero log_10_ *H2* true LRs calculated using STRmix™ (Supplementary material).

**Table 1:** Characteristics of 102 two-person mixture samples analysed using LRmix Studio and STRmix™.

Sample ID	Mixture Type	Sample Type	Receptive Partner drop-outs	POI drop-outs	Total no. of drop-outs	Conservative Pr(*D*)	APH POI (RFU)	Mx Receptive Partner	Mx POI
MF_Swb1_Org	male-female	Swab	0	0	0	0.25	3525	54	46
MF_Swb1_Sil	male-female	Swab	0	0	0	0.25	6094	48	52
MF_Swb1_FTA	male-female	Swab	0	1	1	0.27	840	72	28
MF_Swb2_Org	male-female	Swab	0	0	0	0.25	3900	82	18
MF_Swb2_Sil	male-female	Swab	0	0	0	0.26	8306	53	47
MF_Swb2_FTA	male-female	Swab	7	16	23	0.59	439	33	67
MF_Swb3_Org	male-female	Swab	0	0	0	0.25	4279	80	20
MF_Swb3_Sil	male-female	Swab	0	0	0	0.25	3851	67	33
MF_Swb3_FTA	male-female	Swab	0	2	2	0.29	1395	74	26
MF_Swb4_Org	male-female	Swab	0	0	0	0.25	1433	94	6
MF_Swb4_Sil	male-female	Swab	0	0	0	0.25	8235	51	49
MF_Swb4_FTA	male-female	Swab	0	0	0	0.25	3416	59	41
MF_Swb5_Org	male-female	Swab	0	0	0	0.26	10,886	59	41
MF_Swb5_Sil	male-female	Swab	0	0	0	0.25	3555	49	51
MF_Swb5_FTA	male-female	Swab	0	0	0	0.25	4080	81	19
MF_Stn1a_Org	male-female	Stain	0	0	0	0.26	4178	43	57
MF_Stn1a_Sil	male-female	Stain	5	0	5	0.36	8231	9	91
MF_Stn1b_Org	male-female	Stain	0	0	0	0.25	3686	39	61
MF_Stn1b_Sil	male-female	Stain	11	0	11	0.46	1279	5	95
MF_Stn1c_Org	male-female	Stain	1	0	1	0.27	6788	14	86
MF_Stn1c_Sil	male-female	Stain	8	0	8	0.42	7661	2	98
MF_Stn1d_Org	male-female	Stain	0	0	0	0.25	2565	82	18
MF_Stn1d_Sil	male-female	Stain	0	0	0	0.26	9394	24	76
MF_Stn1e_Org	male-female	Stain	1	0	1	0.28	12,011	16	84
MF_Stn1e_Sil	male-female	Stain	7	0	7	0.4	7538	4	96
MF_Stn2a_Org	male-female	Stain	0	8	8	0.14	349	93	7
MF_Stn2a_Sil	male-female	Stain	0	0	0	0.25	2048	79	21
MF_Stn2b_Org	male-female	Stain	0	9	9	0.16	79	96	4
MF_Stn2b_Sil	male-female	Stain	0	0	0	0.25	2456	78	22
MF_Stn2c_Org	male-female	Stain	0	9	9	0.17	360	95	5
MF_Stn2c_Sil	male-female	Stain	0	0	0	0.25	1541	80	20
MF_Stn2d_Org	male-female	Stain	0	5	5	0.09	405	95	5
MF_Stn2d_Sil	male-female	Stain	0	0	0	0.26	1905	75	25
MF_Stn2e_Org	male-female	Stain	0	2	2	0.29	499	95	5
MF_Stn2e_Sil	male-female	Stain	0	0	0	0.25	2543	59	41
MF_Cdm1_Ext1_Org	male-female	Condom	0	2	2	0.3	1549	79	21
MF_Cdm1_Ext1_Sil	male-female	Condom	0	13	13	0.22	19	97	3
MF_Cdm1_Ext2_Org	male-female	Condom	0	11	11	0.2	263	99	1
MF_Cdm1_Ext2_Sil	male-female	Condom	0	10	10	0.18	188	98	2
MF_Cdm2_Ext1_Org	male-female	Condom	1	7	8	0.12	443	92	8
MF_Cdm2_Ext1_Sil	male-female	Condom	0	4	4	0.06	611	91	9
MF_Cdm2_Ext2_Org	male-female	Condom	0	6	6	0.1	461	94	6
MF_Cdm2_Ext2_Sil	male-female	Condom	0	6	6	0.1	698	91	9
MF_Cdm2_Int2_Org	male-female	Condom	12	0	12	0.49	4931	2	98
MF_Cdm2_Int2_Qia	male-female	Condom	15	0	15	0.53	8861	1	99
MM_Swb1_Org	male-male	Swab	18	15	33	0.59	4155	14	86
MM_Swb1_Sil	male-male	Swab	30	10	40	0.73	86	87	13
MM_Swb1_FTA	male-male	Swab	18	8	26	0.55	296	46	54
MM_Swb2_Org	male-male	Swab	5	1	6	0.23	2453	30	70
MM_Swb2_Sil	male-male	Swab	1	0	1	0.12	3098	14	86
MM_Swb2_FTA	male-male	Swab	5	8	13	0.08	1016	71	29
MM_Swb3_Org	male-male	Swab	5	0	5	0.2	3000	23	77
MM_Swb3_Sil	male-male	Swab	28	7	35	0.69	124	99	1
MM_Swb3_FTA	male-male	Swab	0	0	0	0.12	443	86	14
MM_Swb4_Org	male-male	Swab	24	15	39	0.67	8010	0	100
MM_Swb4_Sil	male-male	Swab	19	0	19	0.47	1448	0	100
MM_Swb4_FTA	male-male	Swab	0	8	8	0.03	319	93	7
MM_Swb5_Org	male-male	Swab	16	0	16	0.42	4470	3	97
MM_Swb5_Sil	male-male	Swab	13	0	13	0.37	8273	2	98
MM_Swb5_FTA	male-male	Swab	0	0	0	0.11	2651	38	62
MM_Stn1a_Org	male-male	Stain	0	0	0	0.11	3128	87	13
MM_Stn1a_Sil	male-male	Stain	0	14	14	0.12	23	99	1
MM_Stn1b_Org	male-male	Stain	0	15	15	0.14	113	99	1
MM_Stn1b_Sil	male-male	Stain	7	30	37	0.46	1	99	1
MM_Stn2a_Org	male-male	Stain	0	0	0	0.11	7268	46	54
MM_Stn2a_Sil	male-male	Stain	8	0	8	0.27	2633	24	76
MM_Stn2b_Org	male-male	Stain	0	0	0	0.11	14,760	39	61
MM_Stn2b_Sil	male-male	Stain	7	0	7	0.25	1436	29	71
MM_Stn2c_Org	male-male	Stain	0	0	0	0.11	17,531	47	53
MM_Stn2c_Sil	male-male	Stain	3	0	3	0.16	5543	13	87
MM_Stn2d_Org	male-male	Stain	0	0	0	0.11	13,485	38	62
MM_Stn2d_Sil	male-male	Stain	5	0	5	0.2	5801	15	85
MM_Stn2e_Org	male-male	Stain	0	0	0	0.11	10,763	32	68
MM_Stn2e_Sil	male-male	Stain	5	0	5	0.21	3986	11	89
MM_Stn2f_Org	male-male	Stain	0	0	0	0.11	16,230	51	49
MM_Stn2f_Sil	male-male	Stain	4	0	4	0.18	2858	13	87
MM_Stn2g_Org	male-male	Stain	0	0	0	0.11	12,728	39	61
MM_Stn2g_Sil	male-male	Stain	9	0	9	0.29	1335	12	88
MM_Stn2h_Org	male-male	Stain	4	0	4	0.18	3068	47	53
MM_Stn2h_Sil	male-male	Stain	16	0	16	0.41	878	6	94
MM_Cdm1_Ext1_Org	male-male	Condom	0	14	14	0.11	90	99	1
MM_Cdm1_Ext2_Org	male-male	Condom	0	16	16	0.15	45	97	3
MM_Cdm2_Ext1_Org	male-male	Condom	0	22	22	0.27	1	100	0
MM_Cdm2_Ext2_Org	male-male	Condom	0	14	14	0.12	105	99	1
SMx_1:19_a	Simulated mixture	Simulated Mixture	0	0	0	0.08	821	97	3
SMx_1:19_b	Simulated mixture	Simulated Mixture	0	0	0	0.06	611	97	3
SMx_1:9_a	Simulated mixture	Simulated Mixture	0	0	0	0.07	1860	94	6
SMx_1:9_b	Simulated mixture	Simulated Mixture	0	0	0	0.08	1485	93	7
SMx_1:4_a	Simulated mixture	Simulated Mixture	0	0	0	0.06	5321	86	14
SMx_1:4_b	Simulated mixture	Simulated Mixture	0	0	0	0.07	2333	85	15
SMx_1:2_a	Simulated mixture	Simulated Mixture	0	0	0	0.08	4766	73	27
SMx_1:2_b	Simulated mixture	Simulated Mixture	0	0	0	0.06	4755	75	25
SMx_1:1_a	Simulated mixture	Simulated Mixture	0	0	0	0.08	8809	56	44
SMx_1:1_b	Simulated mixture	Simulated Mixture	0	0	0	0.08	8423	54	46
SMx_2:1_a	Simulated mixture	Simulated Mixture	0	0	0	0.1	11,006	45	55
SMx_2:1_b	Simulated mixture	Simulated Mixture	0	0	0	0.06	7601	41	59
SMx_4:1_a	Simulated mixture	Simulated Mixture	0	0	0	0.06	10,223	27	73
SMx_4:1_b	Simulated mixture	Simulated Mixture	0	0	0	0.07	11,280	29	71
SMx_9:1_a	Simulated mixture	Simulated Mixture	0	0	0	0.07	17,381	15	85
SMx_9:1_b	Simulated mixture	Simulated Mixture	0	0	0	0.08	15,214	15	85
SMx_19:1_a	Simulated mixture	Simulated Mixture	0	0	0	0.1	14,273	5	95
SMx_19:1_b	Simulated mixture	Simulated Mixture	0	0	0	0.06	12,743	7	93

POI: person of interest; Pr(*D*): probability of drop-out; APH: average peak height; RFU: relative fluorescence units; Mx: mixture proportion.

**Table 2:** *H1* true LRs calculated using LRmix and STRmix™.

Sample ID	LRmix		STRmix™	
	LR	Log_10_ LR	LR	Log_10_ LR
MF_Swb1_Org	5.16E+15	15.71	8.26E+22	22.92
MF_Swb1_Sil	5.16E+15	15.71	9.86E+22	22.99
MF_Swb1_FTA	1.21E+14	14.08	1.24E+16	16.09
MF_Swb2_Org	5.16E+15	15.71	4.43E+20	20.65
MF_Swb2_Sil	4.52E+15	15.65	9.85E+22	22.99
MF_Swb2_FTA	1.36E+05	5.13	3.60E+00	0.56
MF_Swb3_Org	5.16E+15	15.71	8.99E+20	20.95
MF_Swb3_Sil	5.16E+15	15.71	2.34E+18	18.37
MF_Swb3_FTA	2.61E+14	14.42	2.12E+17	17.33
MF_Swb4_Org	5.16E+15	15.71	1.75E+20	20.24
MF_Swb4_Sil	5.16E+15	15.71	1.17E+22	22.07
MF_Swb4_FTA	5.16E+15	15.71	5.55E+22	22.74
MF_Swb5_Org	4.52E+15	15.65	1.32E+21	21.12
MF_Swb5_Sil	5.16E+15	15.71	9.32E+22	22.97
MF_Swb5_FTA	5.16E+15	15.71	1.71E+21	21.23
MF_Stn1a_Org	4.52E+15	15.65	8.45E+22	22.93
MF_Stn1a_Sil	3.97E+15	15.60	5.94E+22	22.77
MF_Stn1b_Org	5.16E+15	15.71	9.55E+22	22.98
MF_Stn1b_Sil	2.32E+15	15.37	5.98E+22	22.78
MF_Stn1c_Org	5.43E+15	15.73	9.91E+22	23.00
MF_Stn1c_Sil	1.84E+15	15.26	9.64E+22	22.98
MF_Stn1d_Org	5.16E+15	15.71	1.82E+21	21.26
MF_Stn1d_Sil	4.52E+15	15.65	6.94E+22	22.84
MF_Stn1e_Org	3.99E+15	15.60	1.01E+23	23.00
MF_Stn1e_Sil	2.32E+15	15.37	8.90E+22	22.95
MF_Stn2a_Org	7.44E+07	7.87	2.67E+05	5.43
MF_Stn2a_Sil	5.16E+15	15.71	8.20E+19	19.91
MF_Stn2b_Org	1.73E+07	7.24	7.17E+04	4.86
MF_Stn2b_Sil	5.16E+15	15.71	3.77E+20	20.58
MF_Stn2c_Org	1.95E+06	6.29	2.73E+02	2.44
MF_Stn2c_Sil	5.16E+15	15.71	1.02E+20	20.01
MF_Stn2d_Org	2.70E+09	9.43	4.79E+08	8.68
MF_Stn2d_Sil	4.52E+15	15.65	2.73E+20	20.44
MF_Stn2e_Org	1.48E+14	14.17	1.95E+12	12.29
MF_Stn2e_Sil	5.16E+15	15.71	1.50E+20	20.18
MF_Cdm1_Ext1_Org	1.64E+14	14.21	1.43E+14	14.16
MF_Cdm1_Ext1_Sil	4.27E+03	3.63	2.11E+03	3.32
MF_Cdm1_Ext2_Org	1.16E+05	5.06	1.14E+01	1.06
MF_Cdm1_Ext2_Sil	1.85E+06	6.27	1.37E+04	4.14
MF_Cdm2_Ext1_Org	6.24E+07	7.80	1.94E+07	7.29
MF_Cdm2_Ext1_Sil	9.22E+09	9.96	8.52E+08	8.93
MF_Cdm2_Ext2_Org	8.38E+08	8.92	1.34E+09	9.13
MF_Cdm2_Ext2_Sil	8.90E+08	8.95	1.11E+09	9.04
MF_Cdm2_Int2_Org	2.00E+15	15.30	5.48E+20	20.74
MF_Cdm2_Int2_Qia	1.14E+15	15.06	3.56E+21	21.55
MM_Swb1_Org	5.13E+08	8.71	5.24E+11	11.72
MM_Swb1_Sil	8.86E+09	9.95	3.63E+09	9.56
MM_Swb1_FTA	9.82E+10	10.99	3.56E+13	13.55
MM_Swb2_Org	4.25E+14	14.63	4.04E+20	20.61
MM_Swb2_Sil	6.29E+15	15.80	1.14E+22	22.06
MM_Swb2_FTA	5.80E+09	9.76	2.51E+13	13.40
MM_Swb3_Org	6.08E+15	15.78	1.01E+22	22.01
MM_Swb3_Sil	2.86E+11	11.46	1.05E+11	11.02
MM_Swb3_FTA	6.19E+15	15.79	6.74E+18	18.83
MM_Swb4_Org	4.82E+08	8.68	2.40E+13	13.38
MM_Swb4_Sil	5.57E+15	15.75	1.14E+22	22.06
MM_Swb4_FTA	2.50E+04	4.40	3.89E+07	7.59
MM_Swb5_Org	7.43E+15	15.87	1.05E+22	22.02
MM_Swb5_Sil	4.66E+15	15.67	1.02E+22	22.01
MM_Swb5_FTA	6.89E+15	15.84	1.82E+21	21.26
MM_Stn1a_Org	6.89E+15	15.84	8.69E+15	15.94
MM_Stn1a_Sil	7.55E+00	0.88	1.23E+02	2.09
MM_Stn1b_Org	2.67E+00	0.43	1.01E+01	1.00
MM_Stn1b_Sil	1.98E-04	−3.70	1.60E+00	0.20
MM_Stn2a_Org	6.89E+15	15.84	1.15E+22	22.06
MM_Stn2a_Sil	3.77E+15	15.58	6.52E+21	21.81
MM_Stn2b_Org	6.89E+15	15.84	1.16E+22	22.06
MM_Stn2b_Sil	5.90E+15	15.77	2.62E+21	21.42
MM_Stn2c_Org	6.89E+15	15.84	1.16E+22	22.06
MM_Stn2c_Sil	5.13E+15	15.71	1.11E+22	22.05
MM_Stn2d_Org	6.89E+15	15.84	9.41E+21	21.97
MM_Stn2d_Sil	1.06E+16	16.02	8.31E+21	21.92
MM_Stn2e_Org	6.89E+15	15.84	1.06E+22	22.03
MM_Stn2e_Sil	9.38E+15	15.97	3.55E+21	21.55
MM_Stn2f_Org	6.89E+15	15.84	1.16E+22	22.06
MM_Stn2f_Sil	4.18E+15	15.62	7.49E+21	21.87
MM_Stn2g_Org	6.89E+15	15.84	1.15E+22	22.06
MM_Stn2g_Sil	5.60E+15	15.75	9.81E+21	21.99
MM_Stn2h_Org	5.52E+15	15.74	8.30E+21	21.92
MM_Stn2h_Sil	2.84E+15	15.45	1.50E+21	21.18
MM_Cdm1_Ext1_Org	1.61E+01	1.21	2.02E+03	3.30
MM_Cdm1_Ext2_Org	3.63E-01	−0.44	1.11E+02	2.05
MM_Cdm2_Ext1_Org	1.58E-02	−1.80	6.01E-01	−0.22
MM_Cdm2_Ext2_Org	3.53E+01	1.55	5.19E+01	1.71
SMx_1:19_a	8.38E+22	22.92	3.92E+19	19.59
SMx_1:19_b	1.00E+23	23.00	2.34E+17	17.37
SMx_1:9_a	9.16E+22	22.96	2.64E+24	24.42
SMx_1:9_b	8.38E+22	22.92	3.52E+21	21.55
SMx_1:4_a	1.00E+23	23.00	4.02E+23	23.60
SMx_1:4_b	9.16E+22	22.96	3.46E+25	25.54
SMx_1:2_a	8.38E+22	22.92	4.41E+25	25.64
SMx_1:2_b	1.00E+23	23.00	1.71E+25	25.23
SMx_1:1_a	8.38E+22	22.92	6.25E+25	25.80
SMx_1:1_b	8.38E+22	22.92	6.26E+25	25.80
SMx_2:1_a	7.04E+22	22.85	6.26E+25	25.80
SMx_2:1_b	1.00E+23	23.00	6.26E+25	25.80
SMx_4:1_a	1.00E+23	23.00	6.26E+25	25.80
SMx_4:1_b	9.16E+22	22.96	6.26E+25	25.80
SMx_9:1_a	9.16E+22	22.96	6.26E+25	25.80
SMx_9:1_b	8.38E+22	22.92	6.26E+25	25.80
SMx_19:1_a	7.04E+22	22.85	6.26E+25	25.80
SMx_19:1_b	1.00E+23	23.00	6.26E+25	25.80

**Table 3:** Summary of *H2* true tests results for LRmix and STRmix™.

Sample ID	LRmix							STRmix™	
	Min	0.01	0.50	0.99	Max	No. of *H2* true LRs > *H1* true LR	No. of *H2* true tests>1	No. of *H2* true LRs > *H1* true LR	No. of *H2* true LRs>1
MF_Swb1_Org	−40.97	−36.01	−25.71	−14.12	−9.01	0	0	0	0
MF_Swb1_Sil	−42.98	−36.51	−25.67	−13.90	−5.88	0	0	0	0
MF_Swb1_FTA	−38.47	−33.84	−23.66	−12.44	−2.62	0	0	0	0
MF_Swb2_Org	−40.41	−36.01	−25.61	−14.41	−7.15	0	0	0	0
MF_Swb2_Sil	−42.44	−35.47	−25.31	−14.20	−6.99	0	0	0	0
MF_Swb2_FTA	−15.40	−12.93	−7.96	−1.93	2.13	0	7	0	0
MF_Swb3_Org	−42.73	−36.38	−25.53	−13.85	−6.15	0	0	0	0
MF_Swb3_Sil	−41.04	−35.95	−25.70	−13.99	−7.01	0	0	0	0
MF_Swb3_FTA	−37.40	−32.14	−22.51	−11.82	−5.20	0	0	0	0
MF_Swb4_Org	−41.83	−36.38	−25.58	−13.84	−6.44	0	0	0	0
MF_Swb4_Sil	−40.73	−36.23	−25.64	−14.19	−7.73	0	0	0	0
MF_Swb4_FTA	−43.76	−36.20	−25.73	−13.99	−6.33	0	0	0	0
MF_Swb5_Org	−40.44	−35.60	−25.28	−13.72	−3.36	0	0	0	0
MF_Swb5_Sil	−41.02	−36.02	−25.75	−14.33	−7.98	0	0	0	0
MF_Swb5_FTA	−40.59	−36.05	−25.72	−14.27	−7.49	0	0	0	0
MF_Stn1a_Org	−41.39	−35.76	−25.26	−13.90	−6.22	0	0	0	0
MF_Stn1a_Sil	−37.23	−31.97	−22.61	−12.25	−6.37	0	0	0	0
MF_Stn1b_Org	−42.36	−36.57	−25.70	−14.09	−6.75	0	0	0	0
MF_Stn1b_Sil	−34.04	−29.78	−20.64	−10.77	−2.93	0	0	0	0
MF_Stn1c_Org	−42.03	−35.53	−25.17	−13.60	−6.79	0	0	0	0
MF_Stn1c_Sil	−36.57	−30.42	−21.53	−11.38	−4.94	0	0	0	0
MF_Stn1d_Org	−41.58	−36.46	−25.64	−14.22	−8.70	0	0	0	0
MF_Stn1d_Sil	−42.51	−35.68	−25.19	−13.91	−6.58	0	0	0	0
MF_Stn1e_Org	−39.25	−34.64	−24.63	−13.21	−8.17	0	0	0	0
MF_Stn1e_Sil	−35.86	−31.17	−21.90	−11.54	−5.26	0	0	0	0
MF_Stn2a_Org	−35.92	−30.77	−20.72	−10.37	−5.26	0	0	0	0
MF_Stn2a_Sil	−41.51	−35.91	−25.67	−14.35	−8.75	0	0	0	0
MF_Stn2b_Org	−34.65	−29.45	−19.92	−9.76	−2.13	0	0	0	2
MF_Stn2b_Sil	−41.83	−35.99	−25.75	−14.00	−3.79	0	0	0	0
MF_Stn2c_Org	−34.30	−28.46	−18.97	−8.78	−1.03	0	0	0	0
MF_Stn2c_Sil	−42.64	−36.13	−25.63	−14.28	−6.53	0	0	0	0
MF_Stn2d_Org	−47.90	−40.61	−28.47	−15.79	−7.55	0	0	0	0
MF_Stn2d_Sil	−40.71	−35.79	−25.35	−14.02	−5.73	0	0	0	0
MF_Stn2e_Org	−38.12	−32.51	−22.53	−12.05	−3.74	0	0	0	0
MF_Stn2e_Sil	−40.81	−36.36	−25.68	−14.17	−8.46	0	0	0	0
MF_Cdm1_Ext1_Org	−34.82	−31.65	−21.91	−11.39	−5.15	0	0	0	0
MF_Cdm1_Ext1_Sil	−26.25	−21.86	−14.03	−5.20	−0.64	0	0	0	7
MF_Cdm1_Ext2_Org	−29.25	−24.07	−15.90	−6.79	−1.09	0	0	1	3
MF_Cdm1_Ext2_Sil	−31.41	−26.45	−17.57	−7.88	0.24	0	1	0	3
MF_Cdm2_Ext1_Org	−41.42	−36.59	−24.85	−12.76	−3.70	0	0	0	0
MF_Cdm2_Ext1_Sil	−53.32	−47.11	−33.34	−18.71	−10.73	0	0	0	0
MF_Cdm2_Ext2_Org	−44.68	−38.81	−26.87	−14.26	−7.42	0	0	0	0
MF_Cdm2_Ext2_Sil	−45.14	−38.61	−26.92	−14.42	−8.18	0	0	0	0
MF_Cdm2_Int2_Org	−33.90	−28.60	−19.87	−10.16	−4.67	0	0	0	0
MF_Cdm2_Int2_Qia	−32.66	−27.40	−18.95	−9.27	−3.48	0	0	0	0
MM_Swb1_Org	−20.97	−17.92	−10.87	−3.39	0.98	0	7	0	0
MM_Swb1_Sil	−20.12	−17.51	−10.88	−3.55	0.49	0	1	0	697
MM_Swb1_FTA	−26.42	−21.79	−13.96	−5.41	0.82	0	1	0	0
MM_Swb2_Org	−42.19	−37.93	−25.84	−13.33	−5.20	0	0	0	0
MM_Swb2_Sil	−52.13	−45.14	−31.40	−17.59	−10.87	0	0	0	0
MM_Swb2_FTA	−53.81	−43.86	−31.19	−17.12	−7.99	0	0	0	0
MM_Swb3_Org	−47.75	−40.03	−28.07	−15.49	−8.70	0	0	0	0
MM_Swb3_Sil	−23.98	−19.58	−12.24	−4.57	1.78	0	3	0	853
MM_Swb3_FTA	−53.68	−44.65	−31.27	−17.16	−7.58	0	0	0	0
MM_Swb4_Org	−19.79	−16.57	−10.16	−3.00	2.19	0	6	0	0
MM_Swb4_Sil	−37.33	−32.06	−21.91	−11.12	−4.16	0	0	0	0
MM_Swb4_FTA	−58.09	−49.92	−35.01	−19.93	−7.31	0	0	0	0
MM_Swb5_Org	−38.85	−33.24	−22.84	−11.64	−2.83	0	0	0	0
MM_Swb5_Sil	−41.56	−34.54	−23.73	−12.21	−6.59	0	0	0	0
MM_Swb5_FTA	−55.63	−45.34	−31.72	−17.52	−6.55	0	0	0	0
MM_Stn1a_Org	−54.18	−45.53	−31.64	−17.78	−8.19	0	0	0	0
MM_Stn1a_Sil	−36.33	−30.49	−19.99	−8.95	−2.01	0	0	17	91
MM_Stn1b_Org	−31.99	−28.08	−18.01	−7.78	−0.74	0	0	0	5
MM_Stn1b_Sil	−8.00	−6.10	−2.90	0.49	2.65	7253	243	2877	4529
MM_Stn2a_Org	−54.04	−45.44	−31.89	−17.65	−10.81	0	0	0	0
MM_Stn2a_Sil	−44.89	−37.79	−26.37	−14.26	−6.41	0	0	0	0
MM_Stn2b_Org	−52.96	−45.44	−31.87	−17.38	−9.80	0	0	0	0
MM_Stn2b_Sil	−45.53	−38.91	−27.37	−15.04	−7.50	0	0	0	0
MM_Stn2c_Org	−52.26	−45.62	−31.87	−18.24	−9.42	0	0	0	0
MM_Stn2c_Sil	−49.97	−42.54	−29.55	−16.40	−6.37	0	0	0	0
MM_Stn2d_Org	−51.69	−44.90	−31.87	−17.63	−9.11	0	0	0	0
MM_Stn2d_Sil	−47.42	−40.79	−28.65	−15.54	−8.42	0	0	0	0
MM_Stn2e_Org	−50.46	−45.19	−31.94	−18.12	−9.68	0	0	0	0
MM_Stn2e_Sil	−48.87	−39.46	−27.36	−14.62	−5.57	0	0	0	0
MM_Stn2f_Org	−50.68	−45.37	−31.85	−17.70	−6.65	0	0	0	0
MM_Stn2f_Sil	−40.90	−41.59	−29.32	−16.31	−9.15	0	0	0	0
MM_Stn2g_Org	−53.92	−45.26	−31.75	−17.86	−10.47	0	0	0	0
MM_Stn2g_Sil	−42.06	−36.75	−25.40	−13.47	−7.04	0	0	0	0
MM_Stn2h_Org	−52.52	−41.63	−29.36	−15.89	−9.79	0	0	0	0
MM_Stn2h_Sil	−40.25	−33.46	−23.04	−11.82	−6.22	0	0	0	0
MM_Cdm1_Ext1_Org	−36.48	−29.95	−19.85	−9.21	−4.12	0	0	0	25
MM_Cdm1_Ext2_Org	−31.09	−25.75	−16.18	−6.54	1.38	2	1	8	180
MM_Cdm2_Ext1_Org	−20.62	−16.51	−9.98	−2.68	1.64	39	4	7917	3984
MM_Cdm2_Ext2_Org	−35.48	−29.62	−18.99	−8.66	−2.35	0	0	11	64
SMx_1:19_a	−71.60	−60.14	−45.59	−29.91	−19.19	0	0	0	0
SMx_1:19_b	−71.65	−63.10	−47.77	−32.21	−22.81	0	0	0	0
SMx_1:9_a	−69.13	−61.60	−46.76	−31.14	−20.43	0	0	0	0
SMx_1:9_b	−72.99	−60.33	−45.87	−30.39	−21.76	0	0	0	0
SMx_1:4_a	−72.58	−63.24	−47.95	−31.72	−20.33	0	0	0	0
SMx_1:4_b	−71.12	−61.80	−46.65	−31.02	−21.72	0	0	0	0
SMx_1:2_a	−71.26	−60.72	−45.77	−30.50	−19.36	0	0	0	0
SMx_1:2_b	−76.62	−63.02	−47.79	−31.81	−22.25	0	0	0	0
SMx_1:1_a	−71.07	−59.78	−45.54	−29.73	−21.46	0	0	0	0
SMx_1:1_b	−68.92	−60.00	−45.70	−30.54	−23.25	0	0	0	0
SMx_2:1_a	−61.20	−56.37	−43.97	−29.71	−25.25	0	0	0	0
SMx_2:1_b	−74.76	−63.15	−47.97	−31.93	−20.58	0	0	0	0
SMx_4:1_a	−70.77	−62.69	−47.92	−31.73	−24.26	0	0	0	0
SMx_4:1_b	−71.67	−62.21	−46.78	−30.80	−21.89	0	0	0	0
SMx_9:1_a	−71.76	−61.80	−46.69	−31.17	−19.78	0	0	0	0
SMx_9:1_b	−67.94	−60.62	−45.88	−30.01	−18.86	0	0	0	0
SMx_19:1_a	−64.29	−57.99	−43.93	−29.18	−17.34	0	0	0	0
SMx_19:1_b	−70.51	−62.95	−47.84	−31.46	−22.29	0	0	0	0

## 2 Experimental design, materials and methods

Ethical clearance was issued by the University of the Philippines Manila Research Ethics Board (UPMREB Code: 2012-321-01). All sample donors provided written informed consent to participate.

Various post-coital specimens (vaginal and anal swabs, undergarment cuttings, internal and external condom swabs) were obtained from a male-female and a male-male pair. Simulated two-person mixtures from a different male-female pair were also prepared at known proportions. Details on sample collection and processing can be found in Ref. [1]. Briefly, DNA samples were amplified using the PowerPlex® 21 system that targets 20 short tandem repeat (STR) loci then separated and detected using the AB® 3500 Genetic Analyzer (Thermo Fisher Scientific). GeneMapper® *ID-X* v.1.2 (Thermo Fisher Scientific) was used to generate and analyze electropherograms. A total of 102 two-person mixtures of variable quality were available for interpretation.

Likelihood ratios were calculated using LRmix Studio v.2.1.3 [2] and STRmix™ v.2.5.11 [3]. LRmix employs a semi-continuous approach incorporating probabilities of drop-out and drop-in Ref. [4], while STRmix™ is a fully continuous system. It models peak height variation [3], [5], exponential degradation [6], [7], drop-in following a gamma distribution [8], and allele-specific stuttering [5], [9], [10]. It also reports mixture proportions according to Clayton and Buckleton [11]. Both use the Balding and Nichols’ equations (recommendation 4.2 of NRC II) as population genetic model [12], [13].

LRmix does not model stuttering, thus stutter filters were applied. However unlabelled peaks on stutter positions where an allele was expected were manually called to avoid inconsistent decisions on assigning short peaks either as allele or stutter. LRs were calculated using a Pr(*D*) determined by the software which results in the lowest LR. For STRmix™, stutter peaks were included in the input files. Other specific parameters used in operating the software can be found in Ref. [1].

All computations used Philippine population allele frequencies [14] and a 0.03 subpopulation correction factor (*θ*) [15]. Calculations were conditioned on the presence of the female or the receptive partner's profile. The following propositions were evaluated:

***H1***: Person A and person B both contributed to the DNA mixture.

***H2***: Person B and an unknown, unrelated person contributed to the mixture.where person A is the POI or penetrative partner contributor to the mixture, while person B is the receptive partner. For the simulated mixtures, Persons A and B are the male and the female sources of DNA, respectively.

We further conducted non-contributor tests [16] for each interpretation system. This was done by replacing the POI 10,000 times with a randomly generated profile (*H2* is true) from Philippine population allele frequencies [14]. LRmix Studio shows for each test the values for the minimum, maximum, as well as the 1st, 50th, and 99th percentiles among 10,000 LRs calculated, while STRmix™ reports all LR values.
